# Supplementary material for: Denosumab Dosage and Tooth Extraction Predict Medication-Related Osteonecrosis of the Jaw in Patients with Breast Cancer and Bone Metastases
Source: Cancers (Basel). 2025 Jul 4;17(13):2242. doi: 10.3390/cancers17132242 (PMC12249028; doi:10.3390/cancers17132242)
Supplement: Supplementary file 1 [file cancers-17-02242-s001.zip › cancers-3711122-supplementary.pdf]

Supplementary Table S1. Multivariable Cox proportional-hazards model for time-to-MRONJ

**Variable**	**HR**	**95 % CI**	**p-value**
Cumulative denosumab doses (per dose)	0.997	0.989–1.005	0.476
Diabetes mellitus (Yes/No)	1.120	0.730–1.719	0.603
Hormone therapy (Yes/No)	1.022	0.607–1.721	0.934
History of tooth extraction (Yes/No)	2.291	1.503–3.493	***<0.001***
Poor oral hygiene (Yes/No)	1.367	0.902–2.073	0.141

Abbreviations: HR > 1 indicates higher hazard of MRONJ; CI, confidence interval
